# TRAM-34, a Putatively Selective Blocker of Intermediate-Conductance, Calcium-Activated Potassium Channels, Inhibits Cytochrome P450 Activity

**DOI:** 10.1371/journal.pone.0063028

**Published:** 2013-05-07

**Authors:** Jay J. Agarwal, Yi Zhu, Qing-Yu Zhang, Alexander A. Mongin, Lindsay B. Hough

**Affiliations:** 1 Center for Neuropharmacology and Neuroscience, Albany Medical College, Albany, New York, United States of America; 2 Wadsworth Center, New York State Department of Health, and School of Public Health, State University of New York at Albany, Albany, New York, United States of America; Tohoku University, Japan

## Abstract

TRAM-34, a clotrimazole analog characterized as a potent and selective inhibitor of intermediate-conductance, calcium-activated K^+^ (IK_Ca_) channels, has been used extensively in vitro and in vivo to study the biological roles of these channels. The major advantage of TRAM-34 over clotrimazole is the reported lack of inhibition of the former drug on cytochrome P450 (CYP) activity. CYPs, a large family of heme-containing oxidases, play essential roles in endogenous signaling and metabolic pathways, as well as in xenobiotic metabolism. However, previously published work has only characterized the effects of TRAM-34 on a single CYP isoform. To test the hypothesis that TRAM-34 may inhibit some CYP isoforms, the effects of this compound were presently studied on the activities of four rat and five human CYP isoforms. TRAM-34 inhibited recombinant rat CYP2B1, CYP2C6 and CYP2C11 and human CYP2B6, CYP2C19 and CYP3A4 with IC_50_ values ranging from 0.9 µM to 12.6 µM, but had no inhibitory effects (up to 80 µM) on recombinant rat CYP1A2, human CYP1A2, or human CYP19A1. TRAM-34 also had both stimulatory and inhibitory effects on human CYP3A4 activity, depending on the substrate used. These results show that low micromolar concentrations of TRAM-34 can inhibit several rat and human CYP isoforms, and suggest caution in the use of high concentrations of this drug as a selective IK_Ca_ channel blocker. In addition, in vivo use of TRAM-34 could lead to CYP-related drug-drug interactions.

## Introduction

Clotrimazole and related azole antimycotic agents are well known inhibitors of cytochrome P450 (CYP) enzymes [1]. CYPs, which are members of a large family of heme-containing oxidases, are key elements of endogenous biosynthetic and signaling pathways involving steroids, prostaglandins, and fatty acid derivatives, and also play essential roles in xenobiotic metabolism [2]. Each CYP has a specific profile of catalytic activities across a number of substrates. These profiles are important for understanding potential drug-drug interactions due to CYP inhibition, as well as induction [3].

Clotrimazole is also a highly potent blocker of intermediate conductance Ca^2+^-activated K^+^ channels (IK_Ca_) [4]. These channels (also known as IK1, SK4, IK_Ca_3.1 or KCNN4) are expressed in various non-excitable cell types throughout the body. IK_Ca_ channels play a vital role in the loss of cellular water [5] as well as the migration of microglia [6] and mast cells [7]. Because of clotrimazole’s potent IK_Ca_ channel blocking activity, this drug has been used clinically for treating several disorders related to abnormal ion channel activity, such as sickle cell disease [8]. However, clotrimazole’s potent anti-CYP activities account for numerous side effects and systemic toxicity [9].

Because of the toxicity of clotrimazole, efforts have been made to develop more selective IK_Ca_ blockers devoid of CYP-related side effects. Wulff et al. [10] characterized TRAM-34 (1-[(2-chlorophenyl) diphenylmethyl]-1*H*-pyrazole), a triarylmethane pyrazole analog of clotrimazole, as a selective and potent blocker of the IK_Ca_ channel (K_d_ = 20 nM). The finding that TRAM-34 did not inhibit CYP activity was the basis for the claimed increase in selectivity [10]. These results suggest that TRAM-34 could have a pharmacological profile similar to that of clotrimazole, without its anti-CYP side effects. Consequently, TRAM-34 is a marketed research tool which is widely used as a highly selective IK_Ca_ blocker in vitro and in vivo (see Discussion). The drug is currently not in human use. Since published studies of TRAM-34’s activity on CYPs are limited to one isoform (human CYP3A4), and recent studies have reported effects of TRAM-34 which are independent of IK_Ca_ [11], we further explored the effects of this compound on several human and rat CYP isoforms.

## Materials and Methods

### Chemicals and Reagents

7-Ethoxy-4-trifluoromethylcoumarin (EFC) was purchased from Invitrogen (Carlsbad, CA). 7-hydroxy-4-trifluoromethylcoumarin (HFC), fluorescein, resorufin, ketoconazole, potassium phosphate monobasic, potassium phosphate dibasic, sodium hydroxide, and DMSO were purchased from Sigma-Aldrich (St. Louis, MO). Dibenzylfluorescein (DBF), 3-cyano-7-ethoxycoumarin (CEC), 3-cyano-7-hydroxycoumarin (CHC), 7-benzyloxy-4-[trifluoromethyl]coumarin (BFC), recombinant CYP microsomes from baculovirus-infected insect cells (Supersomes), NADPH Regenerating System A and NADPH Regenerating System B were purchased from BD Bioscience (Woburn, MA). Acetonitrile (HPLC grade) was purchased from Fisher Scientific (Pittsburg, PA). Clotrimazole was purchased from MP Bioscience (Buxton, UK), TRAM-34 and fluvoxamine were purchased from Tocris Bioscience (Bristol, UK). Lovstatin (LVS, (1*S*,3*R*,7*S*,8*S*,8a*R*)-8-{2-[(2*R*,4*R*)-4-hydroxy-6-oxooxan-2-yl]ethyl}-3,7-dimethyl-1,2,3,7,8,8a-hexahydronaphthalen-1-yl (2*S*)-2-methylbutanoate) was purchased from Cayman Chemical (Ann Arbor, MI). Fluorometric assays were conducted in black Costar 96-well plates (Corning Incorporated, Corning, NY).

### Fluorescence-based CYP Assays

These assays were performed by methods similar to those described by VanAlstine and Hough [12], Stresser et al. [13], Wulff et al. [10], Henderson et al. [14], and Crespi et al. [15], with minor modifications. All incubations were performed in a final volume of 200 µL of 50 mM potassium phosphate buffer at pH 7.4 containing 1% acetonitrile. CYP inhibitor studies were performed according to the parameters of Table 1. Substrate concentrations were chosen to be near known K_m_ values. As described in the references cited, two different NADPH regenerating systems were used depending on the substrates used (Table 1). Inhibitors were added to either “low” NADPH-regenerating system (final concentration of 8.1 µM NADP^+^, 0.4 mM glucose-6-phosphate, 0.4 mM magnesium chloride, 0.2 U/mL glucose-6-phosphate dehydrogenase) or to “high” NADPH-regenerating system (final concentration of 1.3 mM NADP^+^, 3.3 mM glucose-6-phosphate, 3.3 mM magnesium chloride, 0.4 U/mL glucose-6-phosphate dehydrogenase) in a total of 0.1 mL buffer. Samples were pre-incubated at 37°C for the designated times, and the reactions were initiated with the addition of a substrate and enzyme mixtures (Table 1). For assays utilizing “low” regenerating systems, product formation was monitored continuously by a Victor3 1420 Multilabel Plate Counter at the designated wavelengths (Table 1). For the assays utilizing the “high” regenerating system, enzyme reactions were terminated at the designated time by the addition of 75 µL of 2N NaOH, followed by a 2 h post-incubation at 37°C. Product formation was then measured at the designated wavelengths (Table 1). For the “low” regenerating system assays, blanks and standards were prepared in 0.1 mL buffer without the regenerating system. For the “high” regenerating system assays, blanks and standards containing 0.1 mL of buffer received 75 µL of 2N NaOH following pre-incubation. Enzyme activity was linear with incubation time in all assays. TRAM-34 (30 and 100 µM) did not significantly alter the fluorescent signal from any of the CYP products listed in Table 1.

**Table 1:** Conditions for CYP Assays.

CYP	Enzyme (pmol)	Subs (Conc, µM)	Regen. Sys.^a^	Pre-Inc. Time (min)	Inc. Time (min)	Product	Excit/Emiss (nm)
CYP2C6	2	EFC (5)	Low	15	17	HFC	405/535
CYP2C11	1	DBF (0.5)	High	10	30	Fluorescein	485/535
CYP1A2r	1	EFC (112.5)	Low	15	17	HFC	405/535
CYP2B1	1	EFC (37.5)	Low	15	17	HFC	405/535
CYP2C19	1	DBF (0.5)	High	10	30	Fluorescein	485/535
CYP19A1h	1	DBF (0.2)	High	10	30	Fluorescein	485/535
CYP1A2h	0.5	CEC (5)	Low	15	17	CHC	405/460
CYP2B6	1	EFC (2.5)	Low	15	17	HFC	405/535
CYP3A4	1	DBF (1)	High	10	20	Fluorescein	485/535
CYP3A4	2	BFC (50)	Low	15	5	HFC	405/535
CYP3A4^b^	2	LVS (10)	NADPH	15	20	See text	See text

Conditions used for the present CYP assays are summarized. All assays used fluorescence plate reader methods except where noted otherwise.

a See text for regenerating system compositions.

b LC-MS/MS methods used for this assay only.

### CYP3A4 Assay with LVS as Substrate

CYP3A4-containing Supersomes (2 pmol) were incubated with LVS (10 µM**)** in a 200 µl reaction mixture containing 0.1 M potassium phosphate buffer, pH 7.4, 1.0 mM NADPH, and 3 mM MgCl_2_ for 20 min at 37°C. The reaction was initiated by the addition of NADPH, and terminated by the addition of 400 µL of acetonitrile to the reaction mixture. Samples were extracted and analyzed for the two major LVS metabolites 6′β-hydroxy LVS and 6′-exomethylene LVS by LC-MS-MS exactly as described [16].

## Results

### Effects of TRAM-34 on Rat CYP Activity

**Figure 1:**
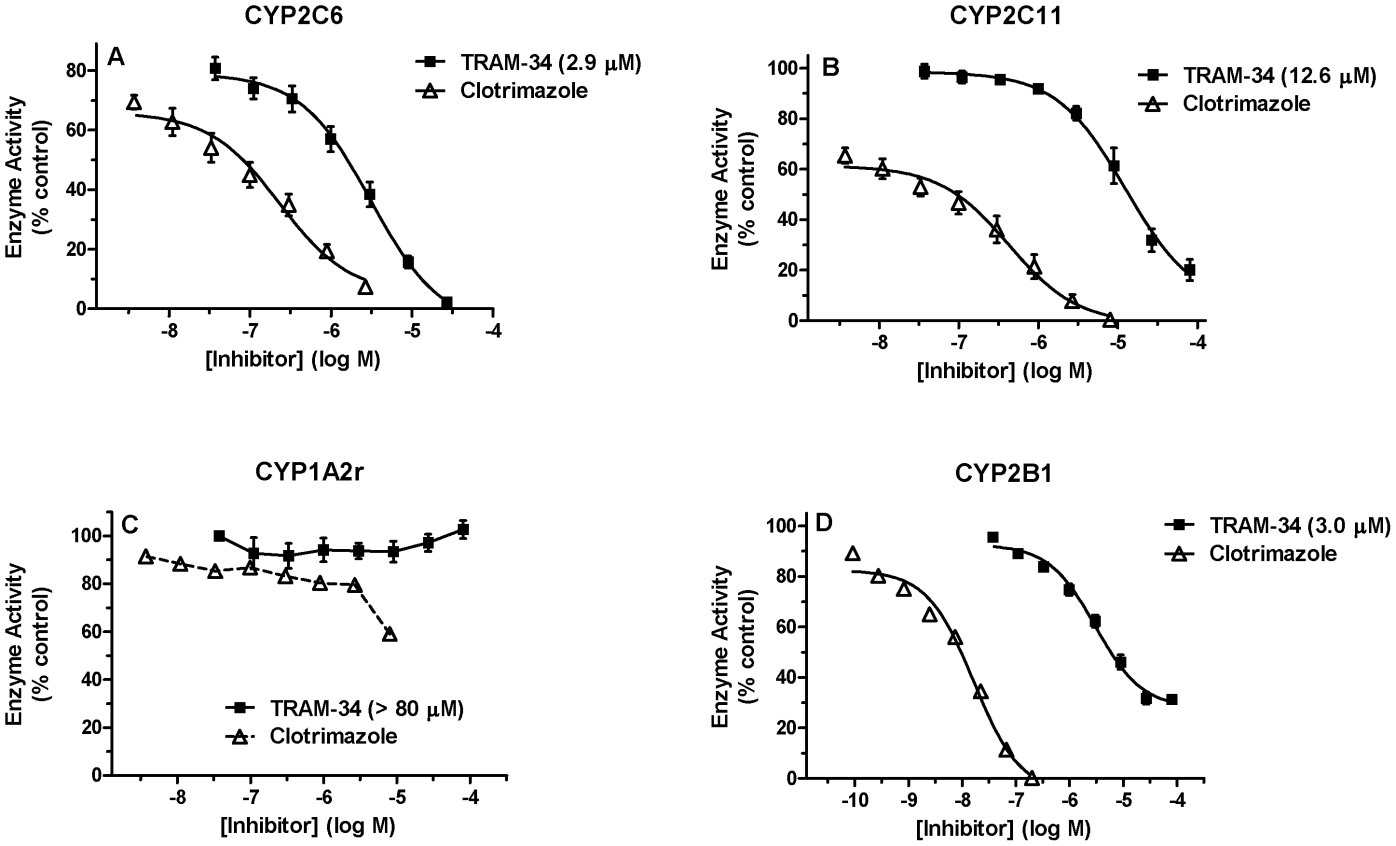
Effects of TRAM-34 on rat CYP Activity. Recombinant enzyme CYP2C6 (A), CYP2C11 (B), CYP1A2r (C) and CYP2B1 (D), substrate and varying concentrations of TRAM-34 were incubated in the presence of 50 mM potassium phosphate buffer and regenerating system at 37°C according to the methods described. Percent control enzyme activity (ordinate) is plotted versus the log of inhibitor concentration (abscissa). All TRAM-34 and clotrimazole data points (A and B) represent the mean (±SEM) of 3 experiments performed in triplicates. Other clotrimazole data represent the mean of duplicates (C) or triplicates (D) from a single experiment. TRAM-34 IC_50_ values were determined by non-linear regression and are shown in parentheses. Control enzyme activities were (mean ± SEM, n = 3 experiments each) 1.65±0.34 (**A**), 0.14±0.02 (**B**), 0.68±0.11 (**C**) and 6.13±0.7 (**D**) min^–1^. In this and subsequent figures, error bars represent SEM of measurements, but, due to the small variability, are not always visible.

TRAM-34 was tested on CYP activities from 4 rat and 5 human isoforms. Surprisingly, concentration-dependent inhibition by TRAM-34 was seen with 3 rat CYP isoforms. TRAM-34 potently inhibited CYP2C6 and CYP2B1 (IC_50_ = 2.9 µM and 3.0 µM respectively, Figs. 1A and 1D). The drug showed weaker inhibition on CYP2C11 with an IC_50_ value of 12.6 µM (Fig. 1B). Clotrimazole, used as a positive control, was a potent inhibitor of CYP2C6, CYP2B1, and CYP2C11, as expected. TRAM-34 showed no inhibition at concentrations up to 80 µM on CYP1A2r (Fig. 1C). Clotrimazole only partly inhibited CYP1A2r activity (Fig. 1C). Other inhibitors (fluvoxamine and miconazole) did not inhibit CYP1A2r activity up to 80 µM (not shown).

### Effects of TRAM-34 on Human CYP Activity

**Figure 2:**
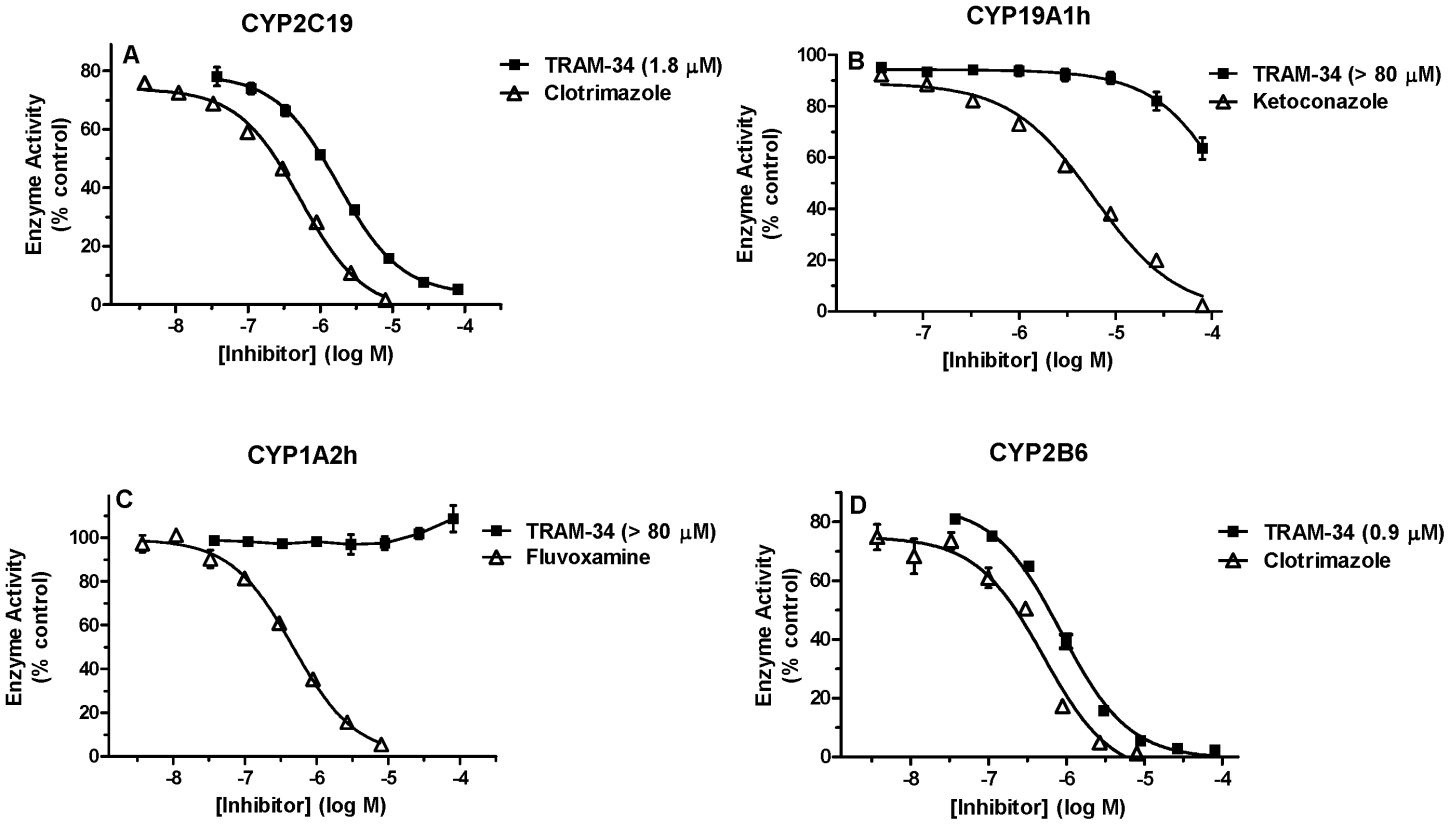
Effects of TRAM-34 on human CYP Activity. Recombinant enzyme CYP2C19 (A), CYP19A1h (B), CYP1A2h (C) and CYP2B6 (D), substrate and varying concentrations of TRAM-34 were incubated in the presence of 50 mM potassium phosphate buffer and regenerating system at 37°C according to the methods described. Percent control enzyme activity (ordinate) is plotted versus the log of inhibitor concentration (abscissa). All TRAM-34 (**A–D**) and fluvoxamine (**C**) data points represent the mean (±SEM) of 3 experiments performed in triplicates. Data from the other inhibitors (A, B and D) represent the mean (±SEM) of triplicates from a single experiment. TRAM-34 IC_50_ values were determined by non-linear regression and are shown in parentheses. Control enzyme activities were (mean ± SEM, n = 3 experiments) 0.30±0.03 (**A**), 0.13±0.003 (**B**), 4.26±0.09 (**C**) and 3.81±0.32 (**D**) min^−1^.

TRAM-34 also showed concentration-dependent inhibition of some human CYP isoforms. The drug potently inhibited CYP2C19 and CYP2B6 (IC_50_ = 1.8 µM and 0.9 µM respectively, Figs. 2A and 2D). Clotrimazole, used as a positive control for CYP2C19 and CYP2B6, was a potent inhibitor of enzyme activities, as expected. TRAM-34 (up to 80 µM) showed little to no inhibition on CYP19A1h or CYP1A2h (Fig. 2B and 2C). Ketoconazole was a potent inhibitor of CYP19A1h activity, as expected. Also, unlike the results with CYP1A2r, fluvoxamine was a potent inhibitor of CYP1A2h (Fig. 2C).

### Effects of TRAM-34 on CYP3A4 Activity

**Figure 3:**
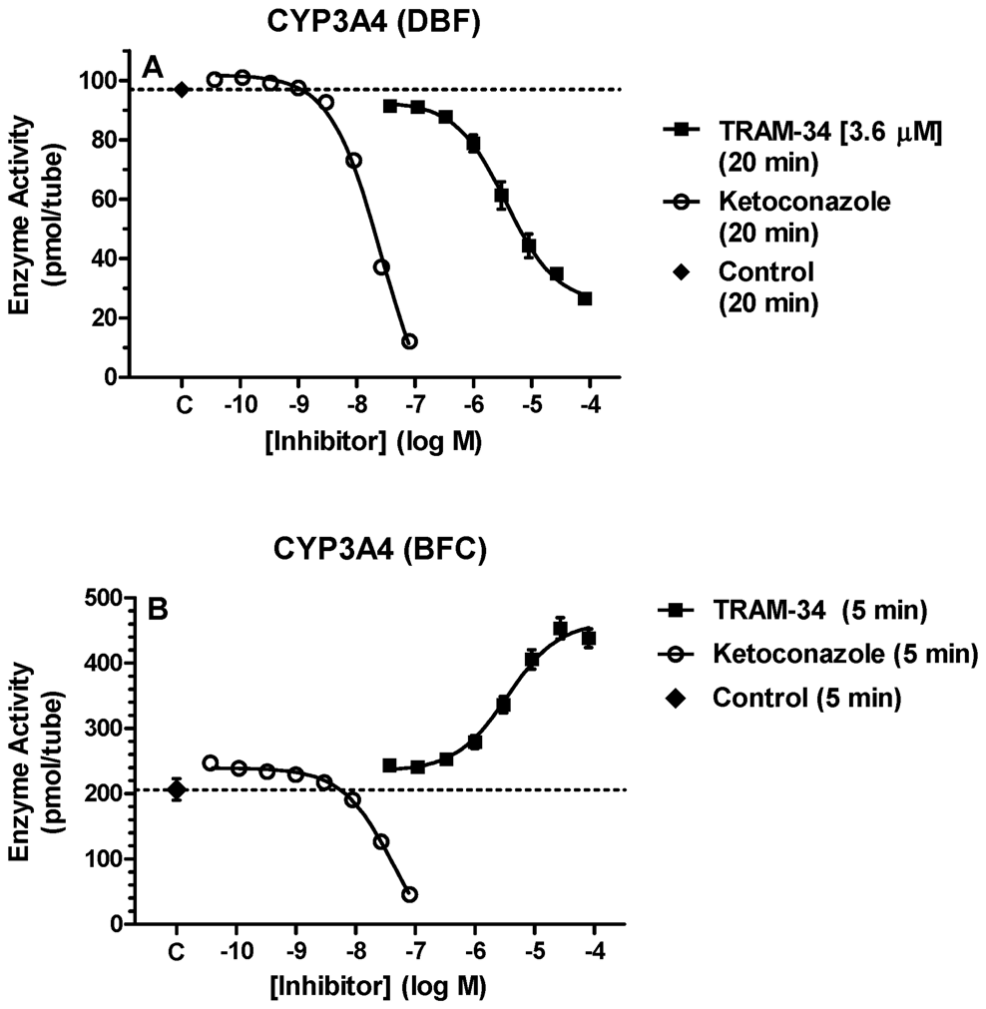
Concentration-dependent inhibition and activation of CYP3A4 by TRAM-34 with two substrates. Recombinant enzyme CYP3A4, substrates DBF (A) or BFC (B) and varying concentrations of TRAM-34 were incubated in the presence of 50 mM potassium phosphate buffer and regenerating system at 37°C according to the methods described. Pmol of product (ordinate) is plotted versus the log of inhibitor concentration (abscissa) for the incubation times specified in parenthesis. All TRAM-34 data points represent the mean ±SEM of 3 experiments performed in triplicate. Data from ketoconazole represent the mean ±SEM of triplicates from a single experiment. The TRAM-34 IC_50_ value was determined by non-linear regression and are shown in brackets (A). Control data points (i.e. no inhibitor, C on abscissa) represent the mean ±SEM from 3 experiments.

TRAM-34 was tested on CYP3A4 with three different substrates. TRAM-34 showed potent and concentration-dependent inhibition of CYP3A4 with DBF (IC_50_ = 3.6 µM, Fig. 3A). Ketoconazole, used as a positive control, was a potent inhibitor of this CYP3A4 activity. Because of the inhibition seen with TRAM-34 in Fig. 3A, TRAM-34 was also tested on CYP3A4 with another substrate, BFC (used previously with CYP3A4 [10]). Surprisingly, TRAM-34 exerted concentration-dependent stimulation of CYP3A4 with BFC. The magnitude of the stimulation was up to ∼200% (Fig. 3B). Ketoconazole, used as a positive control, potently inhibited CYP3A4 activity with BFC (Fig. 3B).

**Figure 4:**
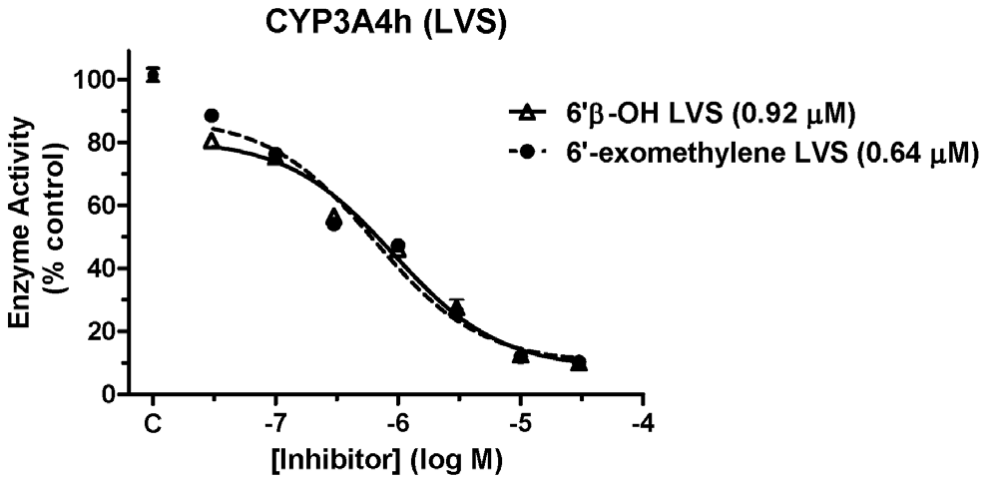
Concentration-dependent inhibition of CYP3A4 by TRAM-34 with LVS as substrate. Recombinant enzyme CYP3A4, LVS and varying concentrations of TRAM-34 were incubated according to the methods described. Percent control enzyme activity (ordinate) is plotted versus the log of inhibitor concentration (abscissa). The formation of two LVS metabolites (in brackets) by CYP3A4 is shown. All TRAM-34 data points represent the mean ± SEM of 3 experiments performed in duplicate. The TRAM-34 IC_50_ values were determined by non-linear regression and are shown in parentheses. Ketoconazole (10 µM) inhibited the formation of both metabolites by greater than 85% (not shown).

CYP3A4 activity was further monitored with the clinically-relevant substrate LVS, an anti-hypercholesterolemia drug (Fig. 4). TRAM-34 demonstrated concentration-dependent inhibition of the formation of two major lovastatin metabolites, 6′β-hydroxy LVS and 6′-exomethylene LVS, with IC_50_ values approximately 1 µM.

## Discussion

TRAM-34 has been used extensively in research as a selective blocker of IK_Ca_ channels [10], [17]. Initial studies reported this drug to have a high selectivity for IK_Ca_ and to lack inhibitory activity against CYP3A4 [10]. Subsequently, TRAM-34 has been used in vitro to study IK_Ca_ channels in many biological roles, including fibroblast proliferation [17], microglia-mediated toxicity [18], tumor growth [19] and epithelial cell membrane function [20]. In vivo studies with this drug suggested the importance of IK_Ca_ channels in experimental autoimmune encephalomyelitis [21], kidney allograft rejection [22], atherosclerosis [23], and restenosis [24]. Because of the importance of TRAM-34 as a research tool, we presently investigated the effects of this drug on several rat and human CYP isoforms.

The present findings document widely divergent effects of TRAM-34 on the activities of various CYP isoforms. The low inhibitory potencies of TRAM-34 on CYP1A2r, CYP1A2h and CYP19A1h (Figs. 1–2) support earlier claims of high selectivity for this drug on IK_Ca_ [10]. However, our findings that TRAM-34 is a potent inhibitor of CYP2B1 and CYP2B6 (IC_50_ values = 0.9–3.0 µM, Figs. 1–2) are the first published results demonstrating significant CYP inhibition by this compound. The inhibition of human and rat CYP2C isoforms by somewhat higher concentrations of drug (IC_50_ values = 1.8 to 12.6 µM, Figs. 1–2) also has consequences for the selectivity of TRAM-34 for IK_Ca_. While conducting the present study, we became aware of an abstract reporting TRAM-34 induced inhibition of CYP2B1, CYP2C6 and CYP3A2r [25]. Reported potencies were in the low micromolar ranges, similar to the present results.

The effects of TRAM-34 on CYP3A4 are interesting because of the importance of this enzyme in drug metabolism [2]. The present results show clear, potent, actions of TRAM-34 on CYP3A4. Wulff et al. [10] reported that TRAM-34 (10 µM) did not inhibit CYP3A4 when the enzyme was assayed with BFC as substrate. Surprisingly, the current findings showed that TRAM-34 produced concentration-dependent *activation* of this enzyme when BFC was used as substrate (Fig. 3B). While the present results are technically in agreement with Wulff et al. [10] (i.e. no CYP inhibition), they clearly demonstrate modulation of CYP3A4 activity by TRAM-34. Wulff et al [10] did not report CYP3A4 activation by TRAM-34, but their data were not shown. Inhibition of CYP3A4 by TRAM-34 was confirmed when either DBF (Fig. 3A) or LVS (Fig. 4) were used as substrates. These results, showing that the same drug can exert opposing actions on CYP3A4 depending on the substrate used (Fig. 3A, 3B and 4), are reminiscent of earlier studies on this enzyme [13]. Such results have been explained by the property of substrate-specific positive cooperativity known to occur with CYP3A4 [26].

Imidazole-containing drugs are well known inhibitors of many CYPs [1]. TRAM-34 was developed by modification of the potent IK_Ca_ blocker and CYP inhibitor clotrimazole [1]. Replacing the imidazole in clotrimazole with a pyrazole led to TRAM-34, which retained the ability to inhibit IK_Ca_ but was reported to not inhibit CYP activity. Although pyrazoles like TRAM-34 have less inhibitory activity on CYPs as compared to clotrimazole, this pyrazole-containing drug remains a CYP inhibitor. Previous studies have also shown some pyrazoles to be even more potent inhibitors of various CYP isoforms than their imidazole congeners [27].

Wulff et al. [10] reported that TRAM-34 is up to 200-fold less potent on other potassium channels (such as the Kv1.2 channel) vs. the IK_Ca_ channel (K_d_ = 20 nM). Our results, showing TRAM-34 modulation of CYP activity in the low micromolar range, suggest a selectivity less than 200-fold for this drug on the IK_Ca_ channel. Current results suggest that *in vitro* concentrations of 0.2–0.8 µM of TRAM-34 would not inhibit the presently studied CYPs, implying 10- to 40- fold selectivity. It should be noted that many additional CYP isoforms exist [28], and should be studied for further evaluation of TRAM-34 selectivity.

The present findings, showing TRAM-34 modulation of CYP activity in the low micromolar range, suggest that some conclusions made by earlier studies using this drug as a selective IK_Ca_ channel blocker may need to be reevaluated. For example, numerous previous *in vitro* studies have used TRAM-34 at concentrations ≥10 µM [20], [29]–[39]. At these concentrations, some CYP isoforms are clear targets of TRAM-34. Previous *in vivo* studies have also used TRAM-34 to inhibit the effects of the IK_Ca_ channel. Although, these studies found plasma concentrations of TRAM-34 to be in the nanomolar range [21], [22], [24], drug concentrations in the liver and subcutaneous-fat 48 h later can be quite substantially higher [24].

The current findings also add to the already developing literature of novel targets for TRAM-34. The drug has been found to inhibit non-selective cation channels [40] and may directly interact with the estrogen receptor in mammary adenocarcinoma cell lines [41]. In addition, Abdullaev et al. [11] found that that the effects of micromolar levels of TRAM-34 on proliferation of malignant cells are completely independent of its effects on IK_Ca_ channels and therefore involve unknown, off-target actions. Our results and these other studies show that TRAM-34 needs to be used with caution even at low micromolar concentrations.

Currently, TRAM-34 is used as an important tool to probe the physiological roles of the IK_Ca_ channel, but the present results suggest that interactions of TRAM-34 with CYPs also need to be considered in both in vitro and in vivo studies. TRAM-34 is not currently used clinically, since a similar triarylmethane drug (ICA-17304, Senicopoc) is in clinical trials for treating sickle cell disease. However, the activities of TRAM-34 on drug-metabolizing enzymes show that in vivo use of this drug for research in animals may produce unexpected drug-drug interactions.
